# Ethical transgressions among healthcare professionals in South Africa from 2014 to 2023

**DOI:** 10.4102/hsag.v30i0.3053

**Published:** 2025-09-19

**Authors:** Maureen A. Pontarelli, Yanet Gezu, Mila Nortjé, Grace Truong, Neela Ravi, Andrew Baldassarre, Willem Hoffmann, Nico Nortjé

**Affiliations:** 1 The Center for Clinical Ethics in Cancer Care, The University of Texas MD Anderson Cancer Center, Houston, United States of America; 2 Department of Informatics, Augustana College, Rock Island, United States of America; 3 Department of Psychology, Christopher Newport University, Newport News, United States of America; 4 Department of Health and Human Physiological Sciences, Skidmore College, Saratoga Springs, United States of America; 5 Department of Sarcoma Medical Oncology, The University of Texas MD Anderson Cancer Center, Houston, United States of America; 6 Department of Philosophy, Houston Community College, Houston, United States of America; 7 Private Practice, Eindhoven, Netherlands; 8 Department of Dietetics and Nutrition, University of the Western Cape, Bellville, South Africa

**Keywords:** ethical transgression, HPCSA, complaints, social media, ethics education

## Abstract

**Background:**

Patients have become more comfortable lodging complaints with professional regulatory bodies over the last several years, likely influenced by the trending nature of patient-centred care and consumerism in healthcare and increased access to information through social media.

**Aim:**

To analyse the frequency and nature of reported ethical transgressions and penalties among registered South African healthcare professionals from 2014 to 2023.

**Setting:**

The study took place in South Africa.

**Methods:**

A list of all sanctioned cases was obtained from the Health Professions Council of South Africa (HPCSA) (data were unavailable for 2019). A mixed-methods approach of quantitative and qualitative content analyses was followed.

**Results:**

The study analysed 1012 ethical transgressions among 452 registered medical practitioners, physiotherapists, psychologists, dietitians, dentists and occupational therapists. Fraudulent conduct accounted for the largest number (*n* = 507 [50.1%]) of transgressions across all professions. The most common penalty imposed was a fine of R1000 to R10000 (26% of total penalties). Both a fine and a suspension were imposed in 82% of cases. Completion of an ethics-based educational course accounted for only 3%.

**Conclusion:**

Fraudulent conduct remains the highest reported ethical transgression among registered healthcare professionals in South Africa. Individualised education that remediates deficiencies in ethical behaviours through analysis and reflection could be beneficial in mitigating ethical misconduct among healthcare professionals.

**Contribution:**

This study provides a comparative analysis of ethical transgressions and penalties among healthcare professionals in South Africa. It also offers insights into necessary areas of improvement of education and training and suggests a different approach to disciplinary action.

## Introduction

Ethical transgressions by healthcare professionals disrupt patients’ perception of and trust in care providers who do act ethically. These transgressions not only impact the professionals themselves and their patients but also their respective specialties. Complaints of professional misconduct have shed light on the changes needed within various healthcare specialties to strengthen trust among the public and healthcare practitioners and to protect the patient–healthcare practitioner relationship. Exploring the frequency and types of reported ethical transgressions provides insight into areas requiring reform within specific healthcare sectors.

Globally, patients have gained comfort in lodging complaints with professional regulatory bodies over the past several years, likely influenced by the trending nature of patient-centred care and consumerism in healthcare, and increased access to information through social media (Park et al. 2024). Both patient-centred care and healthcare consumerism have empowered patients to advocate for themselves within the patient–healthcare professional relationship (Park et al. 2024). It is hypothesised that this empowerment has been further influenced by the accelerated social media use over the past 10 years (Kemp 2015, 2024), which has resulted in an increased frequency of reported ethical transgressions by registered South African healthcare professionals, despite previous recommendations by scholars to address the unethical behaviour (Greenberg et al. 2017; Nortjé & Hoffmann 2016).

Nortjé and Hoffmann reviewed cases of ethical misconduct among registered healthcare practitioners in South Africa between 2007 and 2013 (Nortjé & Hoffmann 2016). The authors provided three recommendations to the Health Professions Council of South Africa (HPCSA) to reduce the frequency of transgressions within various healthcare specialties. Firstly, healthcare systems in fee-for-service societies should adhere to the principles of consumerism, namely transparency, honesty, accountability, competence, analysis of risk and compliance with regulations. Secondly, ethical cognisance should be more than mere compliance with codes of conduct; it should captivate and provoke healthcare practitioners towards reflection and discussion. Thirdly, the rehabilitation process for sanctioned practitioners should encompass a component of ethics education (Nortjé & Hoffmann 2016).

This study provides a retrospective analysis of ethical misconduct cases, sanctioned between 2014 and 2023, against the HPCSA-registered healthcare professionals in South Africa, to ascertain the frequency and nature of these transgressions. The analysis aimed to explore the frequency of occurrence of ethical transgressions in the context of trending patient empowerment, as a result of the accelerated use of social media during the study period. The analysis considered whether further recommendations are needed to address the ethical transgressions of healthcare professionals in South Africa. Determining the frequency and types of ethical transgressions committed by healthcare providers is crucial for informing future medical ethics education. Informative and applicable ethics education for healthcare professionals can aid in strengthening public trust in the healthcare field and protect the patient–healthcare professional relationship by reducing the unethical conduct (Bebeau 2009a, 2009b).

## Research methods and design

The HPCSA is a statutory body that oversees the education and registration of healthcare workers within the country. It ensures that professional and ethical standards are upheld and maintained amongst healthcare professionals, and that any complaints made are investigated and handled appropriately (HPCSA n.d.). The *Health Professions Act 56 of 1974* identifies and sets the scope of each profession within the healthcare sector and thus governs the HPCSA. Accordingly, the 12 professional boards of the HPCSA are tasked with the responsibility for determining profession-specific standards of ethical conduct (HPCSA n.d.).

The HPCSA has an extensive process for handling complaints made against healthcare professionals. Firstly, submitted complaints are screened to determine whether the reported practitioner is registered with the HPCSA and whether there are sufficient details in the allegation to proceed forward. The complaint is then analysed, categorised and filed against the name of the practitioner (HPCSA, 2023b). Secondly, the complaint is referred for either mediation or for preliminary investigation, depending on the nature of the unprofessional conduct (ethical transgression). If the complaint is referred for mediation, a mediator will collaborate with both the practitioner and the complainant to resolve the issue. The case only remains open and goes to preliminary investigation if the two parties cannot reach an agreement. Occasionally, on-site investigations are conducted to establish the facts before sending a notice to the preliminary committee of enquiry (HPCSA, 2023b).

The practitioner will be notified of the complaint if the matter cannot be settled through mediation and proceeds to a preliminary investigation. The practitioner is given an opportunity to respond to the allegation(s) made against them. The response, along with any other relevant information, must be received by the HPCSA within 40 days. If the practitioner fails to respond within the 40-day window, the matter, along with notification of lack of response, is forwarded to the committee of preliminary enquiry (HPCSA, 2023b).

The preliminary committee of enquiry may impose a penalty if the ethical transgression is deemed minor in its severity. The practitioner will receive a notice of the penalty and must accept or reject the charges within 14 days after receiving the notice. The case is finalised and closed only when the practitioner accepts the charges and makes the payment within the 14-day period. The professional conduct enquiry process is referred to if the practitioner rejects the charges, fails to pay within the appropriate window of time or the ethical transgression is deemed serious in its nature (HPCSA, 2023b).

The professional conduct enquiry process is lengthy and limited to cases where the matter has not been previously settled or where the matter is deemed serious by the preliminary committee of enquiry. If a practitioner is found guilty, the committee may impose any of the penalties in section 42(1) of the *Health Professions Act (Act 56 of 1974)*. The complainant may appeal the decision to the Appeals Committee of Council if the professional is not found guilty of misconduct (HPCSA 2023a; 2023b).

This study analyses all the ethical transgression cases that were decided at the level of a full inquiry and that resulted in a sanction between 01 January 2014 and 31 December 2023, among the registered medical practitioners, physiotherapists, psychologists, dietitians, dentists and occupational therapists in South Africa. It is worth mentioning that the data for 2019 were not available because of the lack of publication by the HPCSA; therefore, the analysis covered 9 years. The HPCSA website provides a publicly available formal annual list of all sanctioned professional misconduct cases (HPCSA 2024). Therefore, it was not necessary to obtain formal ethics clearance for this study.

The cases on the HPCSA website are listed chronologically by the month and year in which the sanction was determined. Each case included the practitioner’s name, registration number, nature of the complaint, issued penalty and location. Although the published lists include identifying information for practitioners, such as names and registration numbers, this information is not reported in this article.

The frequencies of ethical transgressions and penalties were analysed by a team of researchers using a quantitative-qualitative sequential design (Draucker et al. 2020). The quantitative component centred on the yearly frequency data of cases per professional category, cases per practitioner, overall counts of general and specific sanctions and overall counts of general and specific penalties. This was followed by the qualitative components, which centre on an archival research approach as an initial point of categorisation (Nortjé & Hoffmann 2016). The listed transgressions were organised into 1 of 9 general categories and 1 of 111 identified specific transgressions (as shown in Table 1-A1). General content descriptions were developed for each specific transgression category; these aided in data analysis and allowed for a review of trends amongst specific forms of misconduct and macro-level transgression clusters (Table 1).

**TABLE 1:** Specific transgressions by transgression category.

Transgression category	Specific transgression
Abuse	Physical abuse Sexual abuse^9^ Verbal abuse
Criminal convictions	Defamation Regulatory or legal contravention Theft
Disclose confidential information without permission	Breach of confidentiality
Fraudulent conduct	Falsifying status or qualification Fraud (advertising material, medical records or reports, financial reports)^2^ Fraudulent billing^1^
Improper professional role conduct	Bribery Failure to respond to the HPCSA enquiry^8^ Unprofessional behaviour^7^ Engaging in a conflict of interest
Negligence or incompetence in evaluating, treating and caring for patients	Abandonment Below-standard medical practice^5^ Below-standard medical practice involving a minor Negligent patient management Negligent medical practice placing patient at unnecessary risk^3^ Negligent medical practice placing patient at unnecessary risk – medication Negligent medical practice placing patient at unnecessary risk – surgical Unsafe or unhygienic work environment
Negligence regarding patient documents or records	Negligent documentation^4^
Perform procedures and interventions without patient consent	Failure to obtain informed consent^10^ Failure to obtain consent for charging above-medical-aid fees Failure to obtain consent for the intervention procedure Failure to obtain parental consent for minor
Professional registration misconduct	Misrepresentation (advertisements) Not compliant with regulatory standards for medical practice and staff^6^ Professional registration misconduct

*Source:* Adapted from, Nortjé, N. & Hoffmann, W., 2016, ‘Seven year overview (2007–2013) of ethical transgressions by registered healthcare professionals in South Africa’, *Health SA Gesondheid* 21, a933. https://doi.org/10.1016/j.hsag.2015.11.004

Note: The overall 10 most frequent transgressions are indicated with superscript numbers (e.g., 1 = most frequent, 2 = second-most frequent).

### Ethical considerations

An application for full ethical approval was made to The University of Texas MD Anderson Cancer Center, and ethics consent was received on 24 March 2025. The ethics waiver number is 2025-0303. The Institutional Review Board issued an ethics waiver for the study because the proposed activity is not research involving human subjects as defined by Department of Health and Human Services (DHHS) and U.S. Food and Drug Administration (FDA) regulations.

## Results

Between 2014 and 2023, there were 1012 ethical transgressions among 452 registered medical practitioners, physiotherapists, psychologists, dietitians, dentists and occupational therapists in South Africa (Tables 2 and Table 3). The greatest number of disciplined healthcare professionals was in 2014 (*n* = 110), while the lowest was in 2023 (*n* = 20). This decrease in the number of sanctioned healthcare professionals can likely be attributed to the transition to telehealth medicine that occurred after the coronavirus disease 2019 (COVID-19) pandemic (Table 2).

**TABLE 2:** Annual number of sanctioned healthcare professionals per professional board (2014 to 2023).†

Professional board	2014	2015	2016	2017	2018	2020	2021	2022	2023	Total
Dentistry	15	6	11	5	2	4	2	2	0	**47**
Medical	85	37	52	23	18	39	27	47	19	**347**
Psychology	8	1	9	2	1	2	6	4	1	**34**
Occupational therapy	1	2	0	0	1	0	3	2	0	**9**
Physiotherapy	1	4	2	2	1	1	1	1	0	**13**
Dietetics	0	0	1	0	0	0	1	0	0	**2**

**Total**	**110**	**50**	**75**	**32**	**23**	**46**	**40**	**56**	**20**	**452**

† , The data for 2019 were not available for analysis.

**TABLE 3:** Total number of sanctioned healthcare professionals (% of mean number of annual professionals) and transgressions per professional for each professional board (2014 to 2023).†

Professional board	Average annual number of healthcare professionals	Total number of sanctioned healthcare professionals	% Mean annual professional	Total number of transgressions	Average number of transgressions per sanctioned healthcare professional
Dentistry	6299	47	0.75	82	1.74
Medical	45 435	347	0.76	655	1.89
Psychology	8491	34	0.40	178	5.24
Occupational therapy	5297	9	0.17	66	7.33
Physiotherapy	7742	13	0.17	26	2.00
Dietetics	3437	2	0.06	5	2.50

**Total**	**76 700**	**452**	**-**	**1012**	**2.24**

† , The data for 2019 were not available for analysis.

Medical practitioners, dentists and psychologists had the highest percentage of sanctioned healthcare professionals when compared to the remaining professions. An average of 0.76% of the 45 435 registered medical practitioners who practised from 2014 to 2023 committed ethical transgressions compared with 0.75% of 6 299 registered dental practitioners and 0.40% of 8 491 registered psychologists. These percentages were substantially higher than those of occupational therapists, physical therapists and dietitians (Table 3). The average amount of transgressions per disciplined healthcare practitioner was 2.24, with occupational therapists (7.33) and psychologists (5.24) being the professions with the highest average number (Table 3).

Repeat offenders in this study were defined as individuals who were sanctioned in different years of the study period, for similar or different offences. Interestingly, repeat offenders were only identified among the psychology, dentistry and medical professions (10%, 4% and 3% of sanctioned professionals, respectively) (Table 4). An analysis showed that none of the repeat offenders received their qualifications after 2010.

**TABLE 4:** Repeat offenders of ethical transgressions during the study period (2014 to 2023).†

Overview	Dentistry	Medical	Psychology
Repeat offenders	2 (4%)	11 (3%)	3 (10%)
Avg # of years between offences	3.5	3.3	1.7
Year of qualification obtained	1993–1995	1975–2007	1990–2009
% of transgressed professionals	4.0	3.0	10.0
Repeated with different offence	1.0	8.0	2.0
Repeated with similar offence	1.0	3.0	1.0

† , The data for 2019 were not available for analysis.

Fraudulent conduct accounted for the largest number (*n* = 507 [50.1%]) of transgressions across all the studied professional groups. In the case of occupational therapy, fraudulent conduct accounted for 92% of the ethical transgressions, followed by physiotherapy (65%), dietetics (60%) and dentistry (60%). Negligence or incompetence in evaluating, treating and caring for patients accounted for the second highest number (*n* = 233 [23.0%]) of transgressions across all of the studied professional groups. This category accounted for 32% of all medical and 20% of all dental ethical transgressions but was very low or not found among the other professional groups. Negligence regarding patient documents or records accounted for the third highest percentage of transgressions per category (*n* = 72 [7.1%]) and was the third highest among medical practitioners (10%). Lastly, professional registration misconduct had the fourth highest frequency (*n* = 63 [6.2%]) of overall transgressions, with medical practitioners accounting for the largest proportion (*n* = 51) as shown in Table 5. Fraudulent conduct, improper professional role conduct and performing procedures and interventions without patient consent were the only three transgression categories that were reported for all professions (Table 5).

**TABLE 5:** Total number (percentage) of transgressions per category for each professional board across the total study period (2014 to 2023).

Professional board	Total # of transgressions	Fraudulent conduct	Professional registration misconduct	Negligence or incompetence in evaluating, treating and caring for patients	Improper professional role conduct	Negligence regarding patient documents or records	Perform procedures and interventions without patient consent	Abuse	Criminal convictions	Disclose confidential information without permission
Dentistry	82	49 (60.0%)	6 (7.0%)	16 (20.0%)	5 (6.0%)	0 (0.0%)	5 (6.0%)	0 (0.0%)	1 (1.0%)	0 (0.0%)
Medical	655	229 (35.0%)	51 (8.0%)	210 (32.0%)	42 (6.0%)	68 (10.0%)	22 (3.0%)	18 (3.0%)	6 (1.0%)	9 (1.0%)
Psychology	178	148 (83.0%)	4 (2.0%)	6 (3.0%)	9 (5.0%)	3 (2.0%)	(2.0%)	1 (1.0%)	0 (0.0%)	4 (2.0%)
Occupational therapy	66	61 (92.0%)	2 (3.0%)	1 (2.0%)	1 (2.0%)	0 (0.0%)	1 (2.0%)	0 (0.0%)	0 (0.0%)	0 (0.0%)
Physiotherapy	26	17 (65.0%)	0 (0.0%)	0 (0.0%)	6 (23.0%)	1 (4.0%)	1 (4.0%)	1 (4.0%)	0 (0.0%)	0 (0.0%)
Dietetics	5	3 (60.0%)	0 (0.0%)	0 (0.0%)	1 (20.0%)	0 (0.0%)	1 (20.0%)	0 (0.0%)	0 (0.0%)	0 (0.0%)

**Total**	**1012**	**507 (50.1%)**	**63 (6.2%)**	**233 (23.0%)**	**64 (6.3%)**	**72 (7.1%)**	**33 (3.3%)**	**20 (2.0%)**	**7 (0.7%)**	**13 (1.3%)**

Penalties imposed on guilty practitioners ranged in nature from a cautionary warning to a fine to suspension from practice. Between 2014 and 2023, the most common imposed penalty was a fine from R1000 to R10 000 (26% of total penalties), followed by a fine from R15 000 to R45 000 (18% of total penalties; total range, R1000 to R210 000). The least common penalty was completion of a non-ethics-based course (0.3% of total penalties). Suspensions ranged from 3 months to 5 years. Sanctioned professionals were both fined and suspended in 82% of cases. Completion of an ethics-based educational course accounted for only 3% of the penalties imposed (Table 6).

**TABLE 6:** Frequency of penalties imposed on sanctioned healthcare professionals across the study period (2014 to 2023).†

Penalty	Total number of imposed penalties	% of overall total number of penalties (*n* = 612)
Fine R1000–R10 000	162	27.0
Fine R15 000–R4000	113	19.0
Suspension 3 months–1 year	79	13.0
Caution or caution and reprimand	69	11.0
Fine R50 000–R100 000	69	11.0
Suspension, 1.5–3 years	65	11.0
Medical ethics course	21	3.0
Suspension, 5 years	12	2.0
Removal from register	10	2.0
Fine R120 000–210 000	4	0.7
Practice under supervision	3	0.5
Community service	3	0.5
Non-ethics course	2	0.3

† , The data for 2019 were not available for analysis.

## Discussion

We identified 1012 ethical transgressions among 452 professionals from 2014 to 2023, as compared with 1437 among 678 in 2007–2013. Like the results of the previous study conducted by Nortjé and Hoffmann (2016), the highest total numbers of sanctioned professionals were among medical and dental practitioners. It should be noted that the sector of practice was not distinguished in this study because of the lack of continuity but remains an opportunity for further exploration.

Fraudulent conduct, including falsifying status or qualification, false advertising material, fraudulent medical records and financial reports and fraudulent billing, remained the predominant complaint against members of these boards. Negligence or incompetence in evaluating, treating and caring for patients was also among the highest for each professional board as well. This form of negligence included abandonment; practising below-standard medical practice with either adults or minors; negligent patient management; negligent medical practice, placing patients at risk, either generally, surgically or with medication and unsafe or unhygienic work environments. These results are similar to those found during the 2007–2013 study period, in which this category ranked second overall.

Financial penalties remained among the most common types of sanction. During the study period, monetary fines alone comprised 57% of the penalties imposed. Often, monetary fines were combined with suspensions, totalling 82% of all imposed penalties. These findings were similar to those of the previous study conducted between 2007 and 2013, in which most penalties consisted of a monetary fine and/or suspension from practice. In contrast to the previous study period, 3% of the total penalties were required completion of a medical ethics course; this type of penalty was not used in 2007–2013.

Between 2006 and 2016, there was a reported 100% increase in the number of complaints submitted to the HPCSA (Greenberg et al. 2017). This increase in lodged complaints has been attributed to the HPCSA distributing pamphlets directing patients how to place complaints, as well as an increase in patients’ awareness of their rights (Greenberg et al. 2017); this increase is also likely because of the increasing use of social media in the modern age and will continue to grow as more people flock to varying platforms to gather information and communicate. In South Africa alone, there were 11.8 million active social media accounts as of January 2015, equating to 22% of the country’s total population (Kemp 2015). In just 9 years, this number grew to 26 million, representing 42.8% of the total population (Kemp 2024).

The growth in social media use over the last 10 or so years has changed how the world shares and receives information (Patrick, Venkatesh & Stukus 2022). These platforms have expanded individuals’ ability to connect with others experiencing similar symptoms or diseases, in addition to establishing connections over other aspects of life (Patrick et al. 2022). While individuals typically make medical decisions in accordance with their own views and values, these are often shaped or influenced by experience, the environment and one’s social circle, either in-person or online (Hajjaj et al. 2010).

Social media influence has impacted the patient–physician relationship in both positive and negative ways. Increased social media use by patients has resulted in the cultivation of autonomy and a sense of empowerment. As a result, reports have indicated better and equal communication between patients and providers and greater efforts towards the cultivation of harmonious patient–physician relationships (Smailhodzic et al. 2016).

While patient autonomy and empowerment are positive results of social media use and have likely contributed to the increase in complaints of ethical transgressions lodged with the HPCSA within the past several years, increased social media use has also resulted in increased exposure to misinformation (Patrick et al. 2022). Misinformation is information that is false, inaccurate or misleading, often causing confusion among lay people. The spread of misinformation is not always intentional but is often a result of cognitive bias, anecdotal stories told in lieu of reported strong scientific evidence, the influence of celebrities and content creators and the sharing of pseudoscience and conspiracy theories (Patrick et al. 2022). The spread of misinformation through increased social media use has resulted in patients reporting suboptimal interactions during healthcare visits and an increase in patients switching who they seek care from (Smailhodzic et al. 2016). Increased social media use and the resultant spread of misinformation have further blurred the line between what is understood as patient-centred care and what is understood as healthcare consumerism.

Healthcare consumerism initially gained popularity because of its rejection of medical paternalism and endorsement of patient autonomy. More recently, it has gained a negative connotation because of its treatment of patients strictly as participants in market transactions (Park et al. 2024). Increased participation in online health communities, often through social media, has aided in empowering patients to advocate for themselves in the patient–physician relationship. However, this increase in participation has also complicated clinical consultations such that professional knowledge and authority are often threatened and the concept of healthcare consumerism is further endorsed (Bernardi & Wu 2022).

It must be acknowledged that increased social media use is likely not the only contributing factor to professional misconduct and the reporting thereof. Factors such as lack of discipline for repeat violations, selfish motivations and lackadaisical oversight by peers, colleagues and boards have been found to directly contribute to varying types of egregious ethical offences by healthcare professionals (DuBois et al. 2018). In addition, several environmental factors have been identified as potentially playing a role in the conduct of unprofessional behaviour, including conflicting roles, financial compensation for wrongdoing, others benefitting from wrongdoing, penalties (to the professional or others) for proper or professional behaviour, mistreatment of the professional, ambiguous or vague professional norms, a population of particularly vulnerable victims, oversight failure and the professional’s authority over their co-workers. These environmental variables have been found to be predictive of wrongdoing in a healthcare setting (Dubois et al. 2012). Not much data exist to aid in explaining how and why these violations occur across healthcare fields; further investigation is necessary to understand these factors (DuBois et al. 2018).

Unethical behaviour by healthcare professionals in a climate of increased social media use and healthcare consumerism negatively impacts public perception of the healthcare field and impedes the development of trust within the patient–physician relationship. Previous recommendations for combating unethical behaviour among healthcare workers in South Africa have proven insufficient in an environment of increasing social media use and healthcare consumerism. Further reforms are needed in specific healthcare sectors to mitigate unethical behaviours and trends among healthcare professionals in South Africa.

Ethics education, when used as part of disciplinary action, has the ability to cultivate individuals’ ethical awareness and restore a sense of professionalism (Bebeau 2009b). We recommend the implementation of an ethics education programme like that developed by the Minnesota Board of Dentistry and Dr. Bebeau. This programme is rooted in Rest’s Four Component Model of Morality, the assertion that all four components of morality—moral sensitivity, moral judgement, moral motivation and moral character—must be activated for moral behaviour to occur (Rest 1983). The programme helps participants identify and address the specific behaviour that led to disciplinary action (Bebeau 2009a, 2009b) through individualised education that remediates deficiencies in ethical behaviours through analysis and reflection in a group setting. Progress must be demonstrated before licensure reinstatement (Bebeau 2009a, 2009b). Furthermore, with the increased use of social media, it could be beneficial to build an online platform for ethics education, which implements the teachings put forth in the education programme. This platform could then be used as a resource and outlet for critical reflection.

### Limitations and future studies

It is worth mentioning that the data reported by the HPCSA is itself an amalgamation of reporting by each of its professional boards. As a result, not all data gathered used consistent terminology. We identified several relatively small inconsistencies in the HPCSA reports, including misidentification of transgressors, double counting of certain transgressions and unexplained missing details about transgressions. We are of the opinion that these inconsistencies were not significant enough to compromise our conclusions or undermine the utility of the HPCSA’s reports. Moreover, the transgression records from 2019 are unavailable, resulting in difficulties establishing a clear picture of the frequency and nature of ethical transgressions just before the COVID-19 pandemic. Another limitation of this study is that only the guilty verdicts, as determined by the professional conduct committee, of medical practitioners, physiotherapists, psychologists, dietitians, dentists and occupational therapists were analysed. Thousands of complaints are submitted to the HPCSA each year, but many of these complaints are handled through mediation or by the preliminary committee of enquiry.

Future studies should focus on how to implement ethics education and explore varying methods and approaches to increasing ethical awareness, with the aim of mitigating the frequency of ethical transgressions among healthcare professionals. These studies should be conducted in a controlled setting and should centre on providing quality education and/or mentorship to healthcare professionals who have committed ethical transgressions. What type of ethics education and how it should be taught should be determined through quality improvement studies, followed by retrospective analyses of the data similar to this one.

## Conclusion and recommendations

Ethical transgressions by healthcare professionals disrupt trust and hinder patient–physician relationships. The frequency and types of complaints lodged with the HPCSA over the past 10 years highlight the need for an ethics education plan that does more than mere check-a-box exercise. An education programme like that developed by the Minnesota Board of Dentistry and Dr. Bebeau would allow for an individualised understanding of deficiencies in ethical behaviours and for reflection on how to act ethically to provide the best possible care to patients (Bebeau 2009a, 2009b). This form of ethics education would aid in strengthening the healthcare sector in South Africa and build trust between the public and healthcare professionals, thereby cultivating and supporting the growth and development of strong patient–healthcare professional relationships.

## Appendix 1

**TABLE 1-A1:** One hundred eleven (111) specific transgressions in micro and macro clusters.

Transgression (general)	Transgression (sub)	Transgression (specific)
**Abuse**	Physical abuse	Applied physical force or assaulted a colleague
		Applied physical force or assaulted a patient
	Sexual abuse	Engaged in a sexual or emotionally intimate relationship with a patient
		Indecent or sexual assault of a patient by touch
		Made inappropriate sexual comments or gestures to a patient
		Sexual harassment of a patient
	Verbal abuse	Verbal assault towards a patient
		Verbal assault towards a colleague
**Criminal convictions**	Defamation	Damage to status or respect of profession – Non-medical criminal act prosecution in court of law
	Regulatory or legal contravention	Unlawful employment of another
		Unlawful possession of medicine
	Theft	Theft
**Disclose confidential information without permission**	Breach of confidentiality	Disclosure of confidential information to a third party without permission
**Fraudulent conduct**	Falsifying status or qualification	Issued certificates to people who have not completed training
	Fraud (advertising material, medical records or reports, financial reports)	False advertising
		Issued a false medical certificate
		Falsely claiming patient treatment rendered on report to another medical professional
		Charged for services not rendered and then bribed patients not to report it to the concerned medical aid scheme
		Making findings without sufficient evidence in a psycho-legal matter
	Fraudulent billing	Charged for medication not dispensed
		Charged for medical services and procedures not rendered
		Fraud – split billing (different invoices to medical aid and patient)
		Use incorrect tariff codes on statement
		Charged medical aid for procedures conducted by a non-medical or unregistered person or laboratory
		Charged medical aid for services rendered to a non-member
		Colluding with unregistered person in respect of medical aid claims
		Dispensed generic medicine and submitted a claim to the medical aid for more expensive medicine
		Medication dispensed differed from what was claimed
		Claimed for services rendered without having consulted a patient
		Failed to obtain informed billing consent of main member
		Misled patients into thinking the consultation was free when it was not
**Improper professional role conduct**	Bribery	Bribery
	Failure to respond to the HPCSA enquiry	Did not respond to the board’s enquiry
		Did not respond to the council’s enquiry
	Unprofessional behaviour	Absen t from scheduled work without permission
		Acted in a manner that could put profession in bad light
		Casting reflection on professional reputation or skill of colleague
		Advertising transgression
		Derogative and/or rude language or action towards patient
		Failure to cooperate with colleagues
		Inappropriate relationship with patient or colleague
		Inappropriately conducted a post-mortem examination
		Suppression of patient medical information
	Engaging in a conflict of interest	Engaging in a conflict of interest
**Negligence or incompetence in evaluating, treating and caring for patients**	Abandonment	Abandonment of patient
		Abandonment of patient, specifically endangering life
		Refusal to attend to a patient
	Below-standard medical practice	Delegation of care duties to lesser qualified person
		Diagnosed without seeing patient in-person
		Not seeing patient, yet compiled a report
		Failed to carry out adequate assessment
		Failed to deliver appropriate or adequate patient care
		Failed to explain nature of illness to patient
		Failed to give proper immunisations
		Failed to inform patient of treatment
		Failed to schedule a follow-up consultation
		Failed to show empathy or compassion
		Failure to communicate patient’s diagnose and treatment with family members
		Failed to report and commission a post-mortem of patient who died of unnatural causes
		Failure to discuss fee structure with patient
		Failed to timely submit account to medical scheme leading to non-payment for services rendered
		Failed to report sexual misconduct
	Below-standard medical practice involving a minor	Failed to deliver appropriate or adequate care to a minor
		Failed to report case of child abuse
	Negligent patient management	Failed beyond doubt to demonstrate that patient required prolonged periods of sick leave
		Failed to communicate or refer subsequent management of patient to general practitioner or specialist
	Negligent medical practice placing patient at unnecessary risk	Failure to keep adequate medical or testing equipment at practice
		Endangered life of consumer
		Failed to admit patient to hospital
		Failed to attend to patient timeously
		Failed to diagnose patient appropriately
		Failed to perform proper diagnostic testing
		Failed to perform an appropriate post-operative assessment
		Failed to perform an appropriate pre-operative assessment
		Failed to appropriately monitor and observe symptoms of a condition
		Failed to respond timeously to medical staff’s request
		Failed to treat complications, leading to death
		Failure to provide standard of care
		Inappropriately discharged a patient
	Poor medical practice placing patient at unnecessary risk – medication	Administered medication when allergy was known
		Administered medication when not indicated
		Failed to accurately dose or administer medication
		Prescribed addictive drug to patient with known history of drug dependence
		Prescribed expired medication
		Prescribed incorrect medication
	Poor medical practice placing patient at unnecessary risk – surgically	Failed to monitor patient in recovery room and properly discharge
		Failed to perform the appropriate surgical procedure
		Performing obsolete surgical procedures
	Unsafe or unhygienic work environment	Not wearing appropriate personal protective equipment (PPE)
		Failed to carry out proper hygienic practices
		Failure to keep proper records
**Negligence regarding patient documents or records**	Negligent documentation	Issued medical certificate or prescription without examining or diagnosing the patient
		Issued medical certificates that did not comply with the HPCSA guidelines
		Failed to keep medical certificate or prescription book in a safe place
		Failed to provide medical report
**Perform procedures and interventions without patient consent**	Failure to obtain consent	Failure to inform patient of potential treatment risks and complication
		Failure to obtain patient consent for medical examination
		Failure to obtain patient consent for performed service
	Failure to obtain consent for charging above-medical-aid fees	Failure to inform patient of medical aid scheme’s non-payment for services rendered
		Neglected to obtain patient consent for above-medical-aid expenses
	Failure to obtain consent for intervention procedure	Failed to get informed consent for procedure
	Failure to obtain parental consent for minor	Failed to obtain informed consent from parent for medical treatment of a minor
**Professional registration misconduct**	Misrepresentation (advertisements)	Advertise as a healthcare professional when not registered
		Advertisement in contravention with the HPCSA rules
	Not compliant with regulatory standards for medical practice and staff	Allow or employ an unregistered person to practise as a healthcare professional
		Run a healthcare practice while not registered
		Practice as healthcare professional at an unapproved facility
		Shared premises with unregistered person
	Professional registration misconduct	Practice as a private practitioner while only registered for public health service
		Practice as a healthcare professional when not registered
		Practice under the HPCSA registration number of another healthcare practitioner while unregistered

## References

[CIT0001] Bebeau, M.J., 2009a, ‘Enhancing professionalism using ethics education as part of a dental licensure board’s disciplinary action. Part 1. An evidence-based process’, *Journal of the American College of Dentists* 76, 38–50. 19743688

[CIT0002] Bebeau, M.J., 2009b, ‘Enhancing professionalism using ethics education as part of a dental licensure board’s disciplinary action. Part 2’, Evidence of the process’, *Journal of the American College of Dentists* 76, 32–45. 19928366

[CIT0003] Bernardi, R. & Wu, P.F., 2022, ‘Online health communities and the patient-doctor relationship: An institutional logics perspective’, *Social Science & Medicine* 314, 115494. 10.1016/j.socscimed.2022.115494 36334494

[CIT0004] Draucker, C.B., Rawls, S.M., Vode, E. & Carter-Harris, L., 2020, ‘Integration through connecting in explanatory sequential mixed method studies’, *Western Journal of Nursing Research* 42(12), 1137–1147. 10.1177/0193945920914647 32389099 PMC8355866

[CIT0005] Dubois, J.M., Anderson, E.E., Chibnall, J.T., Diakov, L., Doukas, D.J., Holmboe, E.S. et al., 2018, ‘Preventing egregious ethical violations in medical practice: Evidence-informed recommendations from a multidisciplinary working group’, *Journal of Medical Regulation* 104(4), 23–31. 10.30770/2572-1852-104.4.23 30984914 PMC6461379

[CIT0006] Dubois, J.M., Carroll, K., Gibb, T., Kraus, E., Rubbelke, T., Vasher, M. et al., 2012, ‘Environmental factors contributing to wrongdoing in medicine: A criterion-based review of studies and cases’, *Ethical Behavior* 22(3), 163–188. 10.1080/10508422.2011.641832 PMC3515073 23226933

[CIT0007] Greenberg, M., Redmond, C., Kailasanathan, A., Stephen, I., Lalanda, M., White, P. et al., 2017, *The cost of rising claims and complaints*, p. 25, Medical Protection.

[CIT0008] Hajjaj, F.M., Salek, M.S., Basra, M.K. & Finlay, A.Y., 2010, ‘Non-clinical influences on clinical decision-making: A major challenge to evidence-based practice’, *Journal of the Royal Society of Medicine* 103(5), 178–187. 10.1258/jrsm.2010.100104 20436026 PMC2862069

[CIT0009] HPCSA, n.d., *About us: History*, HPCSA, viewed 08 December 2024, from https://www.hpcsa.co.za/about-us.

[CIT0010] HPCSA, 2023a, *Board committees*, HPCSA, viewed 24 March 2025, from https://hpcsa.co.za/board/environmental-health/board-committees.

[CIT0011] HPCSA, 2023b, *HPCSA complaints process explained*, HPCSA, viewed 24 March 2025, form https://www.hpcsa-blogs.co.za/hpcsa-complaints-process-explained/#:~:text=The%20complaints%20go%20through%20a,analysis%20and%20categorisation%20takes%20place.

[CIT0012] HPCSA, 2024, *Publications*, HPCSA, viewed 10 July 2024, from https://www.hpcsa.co.za/?contentId=338&actionName=Publications.

[CIT0013] Kemp, S.K.W.A.S.M., 2015, *Digital 2015: South Africa*, DataReportal, viewed 18 November 2024, from https://datareportal.com/reports/digital-2015-south-africa.

[CIT0014] Kemp, S.K.W.A.S.M., 2024, *Digital 2024: South Africa*, DataReportal, viewed 18 November 2024, from https://datareportal.com/reports/digital-2024-south-africa.

[CIT0015] Nortjé, N. & Hoffmann, W., 2016, ‘Seven year overview (2007–2013) of ethical transgressions by registered healthcare professionals in South Africa’, *Health SA Gesondheid* 21, a933. 10.1016/j.hsag.2015.11.004

[CIT0016] Park, S.Y., Cook, D.M., Yun, G.W. & Coppes, M.J., 2024, ‘Are patient-centered Care, healthcare consumerism, and trust in physicians compatible?: Positioning analysis of the patient-provider relationship’, *Health Communication* 40(8), 1–11. 10.1080/10410236.2024.2408065 39324998

[CIT0017] Patrick, M., Venkatesh, R.D. & Stukus, D.R., 2022, ‘Social media and its impact on health care’, *Annals Allergy Asthma & Immunology* 128(2), 139–145. 10.1016/j.anai.2021.09.014 34555532

[CIT0018] Rest, J.R., 1983, ‘Morality’, in P.H. Mussen, J. Flavell & E. Markman (eds.), *Handbook of child psychology- cognitive development*, 4th edn., pp. 556–628, John Wiley, New York, NY.

[CIT0019] Smailhodzic, E., Hooijsma, W., Boonstra, A. & Langley, D.J., 2016, ‘Social media use in healthcare: A systematic review of effects on patients and on their relationship with healthcare professionals’, *BMC Health Service Research* 16, 442. 10.1186/s12913-016-1691-0 PMC5000484 27562728

