# Corrigendum: Ethical transgressions among healthcare professionals in South Africa from 2014 to 2023

**DOI:** 10.4102/hsag.v30i0.3350

**Published:** 2025-11-27

**Authors:** Maureen A. Pontarelli, Yanet Gezu, Mila Nortjé, Grace Truong, Neela Ravi, Andrew Baldassarre, Willem Hoffmann, Nico Nortjé

**Affiliations:** 1 The Center for Clinical Ethics in Cancer Care, The University of Texas MD Anderson Cancer Center, Houston, United States of America; 2 Department of Informatics, Augustana College, Rock Island, United States of America; 3 Department of Psychology, Christopher Newport University, Newport News, United States of America; 4 Department of Health and Human Physiological Sciences, Skidmore College, Saratoga Springs, United States of America; 5 Department of Sarcoma Medical Oncology, The University of Texas MD Anderson Cancer Center, Houston, United States of America; 6 Department of Philosophy, Houston Community College, Houston, United States of America; 7 Private Practice, Eindhoven, Netherlands; 8 Department of Dietetics and Nutrition, University of the Western Cape, Bellville, South Africa

In the published article, Pontarelli, M.A., Gezu, Y., Nortjé, M., Truong, G., Ravi, N., Baldassarre, A. et al., 2025, ‘Ethical transgressions among healthcare professionals in South Africa from 2014 to 2023’, *Health SA Gesondheid* 30(0), a3053. https://doi.org/10.4102/hsag.v30i0.3053, the authors neglected to include the funder, Cancer Prevention and Research Institute of Texas (CPRIT) – Research Training Award, CPRIT Training Program, RP210028.

Instead of:

## Funding information

The authors received no financial support for the research, authorship, and/or publication of this article.

It should be:

## Funding information

The authors disclosed receipt of the following financial support for the research. This work was supported by the Cancer Prevention and Research Institute of Texas (CPRIT) – Research Training Award, CPRIT Training Programme (grant number: RP210028).

The authors apologise for this error. The correction does not change the study’s findings, its significance or overall interpretation of the study’s results or the scientific conclusions of the article in any way.

